# Comparison Pregnancy Outcomes Between Minimal Stimulation Protocol and Conventional GnRH Antagonist Protocols in Poor Ovarian Responders

**Published:** 2016-03

**Authors:** Shamim Pilehvari, Ensieh ShahrokhTehraninejad, Batool Hosseinrashidi, Fatemeh Keikhah, Fedyeh Haghollahi, Elham Aziminekoo

**Affiliations:** Reproductive Health Research Center, Tehran University of Medical Sciences, Tehran, Iran

**Keywords:** In Vitro Fertilization, Conventional Antagonist Protocol, Minimal Stimulation Protocol, Poor Ovarian Responders

## Abstract

**Objective:** To compare the pregnancy outcomes achieved by in vitro fertilization (IVF) between minimal stimulation and conventional antagonist protocols in poor ovarian responders (PORs).

**Materials and methods:** In this randomized controlled trial, 77 PORs undergoing IVF were selected and divided into two groups. First group was the minimal stimulation group (n = 42) receiving 100 mg/day clomiphene citrate on day 2of the cycle for 5 day that was followed by150IU/day human menopausal gonadotropin (hMG) on day 5 of the cycle. Second group was the conventional group (n = 35) receiving at least 300 IU/daygonadotropin on day 2 of the cycle. Gonadotropin-releasing hormone (GnRH) antagonist protocol was applied for both groups according to flexible protocol. Number of retrieved oocytes and chemical pregnancy rate were the main outcomes.

**Results:** There was no difference in number ofretrieved oocyte and pregnancy rate (2.79 ± 1.96 vs. 2.20 ± 1.71 and 5.6% vs. 4.1%; p > 0.05) between both groups. The gonadotropin dose used in the minimal stimulation group was lower than conventional group (1046 ± 596 vs. 2806 ± 583).

**Conclusion:** Minimal stimulation protocol with lower gonadotropin used is likely to be considered as a patient- friendly and cost-effective substitute for PORs.

## Introduction

Poor ovarian responders (PORs) are the group of infertile population who is characterized by diminished ovarian reserve and decreased follicular response, resulting in few retrieved oocytes, few transferred embryos, high cancellation rates of cycles, and low clinical pregnancy rates (-). This group was first identified in 1983 (1). The European Society of Human Reproduction and Embryology (ESHRE) then tried to reach a consensus described the Bologna criteria (2011) (4). In spite of great progression in assisted reproductive technology (ART), successful treatment in this group has remained a major challenge in ART programs, and effective therapy for PORs is yet unknown (2, 5, 6). PORs are shown to be related to advanced age, ovarian surgery, endometriosis, chemotherapy, radiotherapy, chronic smoking,genetic factor and iatrogenic causes (7-9). Incidence of PORs has been reported in 9-24% (10, 11). A number of studies have indicated that they need more gonadotropin for stimulation (12). Several interventions were used to improve effectiveness of applied technique and the pregnancy outcomein this group, but there is no adequate evidence which intervention is appropriate (13-15). The most common protocol used for PORs is gonadotropin-releasing hormone (GnRH) agonist (16, 17). Minimal ovarian stimulation is anther protocolthat consists of low dose gonadotropin overlapping clomiphene or letrozole. The main advantages of this protocol are as follows: (i) cost effectiveness, (ii) shorter duration of stimulation, (iii) reduced gonadotropin requirements and (iv) patient-friendly method (3,    18 - 21). However, in some studies, minimal stimulation protocol was not considered as a cost effective and better method in compared with the standard protocols, and it was not also recommended (16, 20, 21). Clomiphene citrate overlapping gonadotropin with GnRH antagonist has been alsoapplied for PORs (22). In a study by Saadat et al., they have shown that clomiphene citrate causesan increase in endogenous follicle stimulating hormone (FSH) level(23) that leads toa low dose of gonadotropin and shorter duration of stimulation as compared with GnRH agonist (19, 22, 24). Therefore, clomiphene combined with gonadotropin offer an advantage for PROs (25). For the purpose of minimum effect on the endometrium, it has been suggested that the protocol starts on the second day of cycle (26). In a study by Ubaldi et al., they have showed that the GnRH antagonist prevents premature luteinizing hormone (LH) surge and ovulation advantages has to compare the GnRH agonist (9). The objective of this study was to compare the pregnancy outcomes achieved by in vitro fertilization (IVF) between conventional antagonist protocoland minimal stimulation protocol including clomiphene citrate overlapping with gonadotropin in PORs.

## Materials and methods

In this randomized controlled trial, 77PORs who were admitted to the Vali-e-Asr Infertility Clinic of Tehran University of Medical Sciences, Tehran, Iran, from March 2014 to June 2015, were selected according to the Bologna criteria. The study was confirmed by the Ethics Committee of Tehran University of Medical Sciences (Ethics code: 9211400001) (Trial number: IRCT2015011920351N2).

At least two of the following three criteria must be present to include the patients: (i) advanced maternal age (≥ 40), (ii) previous PORs (presence of less than 3 oocytes when using a conventional stimulation protocol), and (iii) an abnormal ovarian reserve test [antral follicle count (AFC) < 5-7 follicles or anti-mullerian hormone (AMH) < 0.5-1.1 ng/ml]. 

The exclusion criteria were use of any infertility medicine since last 3 month and presence of any medical history.

Patients were randomly divided into two following groups: minimal stimulation group as study group (n = 42) and conventional antagonist group as control group (n = 35). Furthermore all patients had to sign an informed consent before entering the study.

In minimal stimulation group (study group), patients received 100 mg/day clomiphene citrate (Iran Hormone Co., Iran) on day 2 of the cycle for five daysthat was followed by 150IU/dayhuman menopausal gonadotropin (hMG; Merional; IBSA,Lugano, Switzerland) on day 5 of the cycle (day 4 of clomiphene). Subsequently patients received 0.25 mg/day GnRH antagonist (Cetrotide; Cetrotide; Merck-Serono, Germany) according to flexible protocol when at least one follicle or more reached a mean diameter of 13-14 mm and was continued daily until the day of human chorionic gonadotropin (hCG; Choriomon; IBSA, Lugano, Switzerland) injection.Cycle monitoring was started on day 7 or8 of the cycle using a trasvaginal ultrasound and repeated every 2-3 days. When the mean diameter of the follicle reached 17-18mm, an intramuscular (IM) injection of 10000IU hCG (IBSA) was administered for maturation of follicles. In conventional antagonist group (control group), patients received 300IU gonadotropin (hMG& Gonal-F) from day 2 of the cycle, and its concentration was adjusted by ovarian response every 3-4 days. Then 0.25 mg/day GnRH antagonist (MerckSerono SA) was started according to flexible protocol when at least one follicle reached a mean diameter of 13-14 mm and was continued daily until the day of hCG injection. When the mean diameter of two follicles reached ≥ 17-18 mm, an IM injection of 10000IU hCG (IBSA) was administered. About 34-36 hours after hCG, oocytes were retrieved. Good quality embryos were transferred three days after oocyte retrieval.For luteal phase support, both group received 400progesterone suppositories (Cyclogest; Actover, USA) twice a day that was started on the same day of oocyte retrieval. In both groups, after 14 days of embryos transfer, βhCG level was determined. Number of oocytes retrieved and chemical pregnancy rate were the main outcomes. 

Data analysis was performed using Statistical Package for Social Sciences16.0 (SPSS; SPSS Inc., USA) software.A student's t test was used to determine the differences between the means of two groups. A p-value of less than 0.05 was expressed statistically significance.

## Results

**Figure 1:**
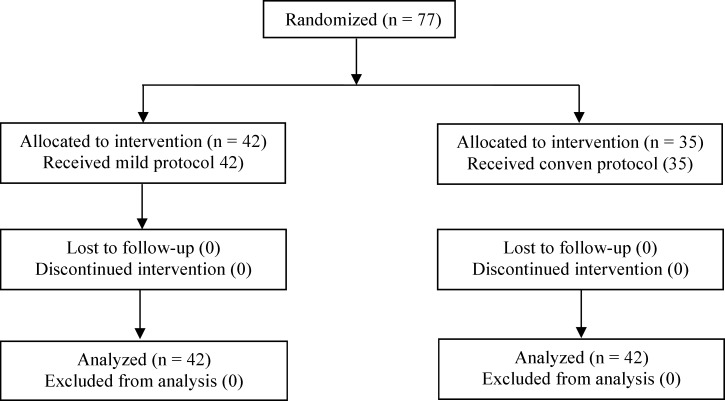
Flow diagram

The results were published inconformity with CONSORT statement (Figure 1). Seventy-seven patients according to Bologna criteria were included in our study, of whom 42 patients were recruited in the study group and 35 in control group. The findings revealed that there were no significant differences between both groups regarding age, body mass index (BMI), duration of infertility, as well as basal FSH and AMH levels (Table 1). There were no statistically significant differences between two groups regarding duration stimulation (9.24 ± 2.21 vs. 9.37 ± 1.83), number of oocytes retrieved (2.20 ± 1.71 vs. 2.79 ± 1.96), and endometrial thickness (8.04 ± 1.88 vs. 8.7 ± 2.3; p > 0.05) (Table 2). Cancellation and pregnancy rates were also similar between two groups (28.6% vs. 31.4%; p > 0.05 and 4% vs.5.6 %; p > 0.05, respectively). Fertilization rate did not differ significantly in both group(66.6 ± 37.7 vs.62.3 ± 34.4; p > 0.05). Total doses of gonadotropin were lower in the study group as compared to the control group (1046 ± 596 vs. 2806 ± 583; p < 0.05).

## Discussion

Different protocols have been used for stimulation of PORs, although there is no consensus about the best protocol (13-15).

The purpose of the study was to compare pregnancy outcomes between minimal stimulation and conventional protocols in PORs. Our results showed that number of oocytes retrieved, duration stimulation and clinical pregnancy rate of minimal stimulation protocol were similar to those of conventional protocol.

**Table 1:** Demographic characteristic of patients

Variables	Minimal stimulation	Conventional antagonist	p value
**Age**	**40.64 ± 4.86**	**38.89 ± 3.88**	**0.09**
**BMI (kg/m²)**	**26.57 ± 3.53**	**25.51 ± 4.52**	**0.48**
**Basal FSH (IU/l)**	**10.55 ± 5.51**	**8.53 ± 5.43**	**0.11**
**Basal LH (IU/l)**	**7.36 ± 8.62**	**5.37 ± 4.42**	**0.22**
**AMH (mmol/l)**	**0.48 ± 0.61**	**0.64 ± 0.47**	**0.19**
**Duration of infertility**	**5.82 ± 5.40**	**7.62 ± 5.75**	**0.16**

BMI: Body mass index; FSH: Follicular stimulating hormone; LH: Luteinizing hormone; AMH: Anti mullerian hormone

**Table 2:** Results of ovarian stimulation using minimal stimulation and conventional protocols

Variables	Minimal stimulation	Conventional antagonist	p value
**Days of stimulation**	**9.24 ± 2.21**	**9.37 ± 1.83**	**0.77**
**Total dose of gonadotropin (IU)**	**1046 ± 596**	**2806 ± 583**	**0.00**
**Endometrial thickness day hCG (mm)**	**8.04 ± 1.88**	**8.77 ± 2.3**	**0.17**
**Number of retrieved oocytes**	**2.2 ± 1.71**	**2.79 ± 1.96**	**0.17**
**Number of transferred embryos**	**1.64 ± 0.81**	**1.74 ± 0.73**	**0.54**
**Cycle cancellation**	**12(28.6%)**	**11 (31.4%)**	**0.78**
**Clinical pregnancy rate (%)**	**1(4%)**	**1 (5.6%)**	**0.05**
**Fertilization rate (%)**	**66.3%**	**62.3 %**	**0.6**
**Quality of embryo (n (%))**			
** A**	**24 (58.54)**	**23(71.8)**	**> 0.05**
** AB**	**12 (29.27)**	**6 (18.75)**	
** B**	**5 (12.19)**	**3 (9.38)**	

Althoughthe dose of gonadotropin overlapping clomiphene or letrozole was lower in minimal stimulation group. The cancellation and fertilization ratesshowed no significant differencesbetween two groups. Different studies have showed that minimal ovarian stimulation protocol has the following advantages: cost effectiveness, shorter duration of stimulation, reduced gonadotropin requirements and patient-friendly method (3, 18-21). It is noteworthy that the studies showed different findings when comparing minimal stimulation protocol with others protocols in PORs. A number of studies also compered mild stimulation protocol with other protocols in PORs (18, 19, 22, 27). Different controlled ovarian hyper stimulation (COH) protocols have also been proposed and used for enhancing pregnancy outcomes in this group (28, 29), including gonadotropin-releasing hormone (GnRH) agonist consisting of long, short, miniflare, super long, modified superlong (22, 28, 30) and GnRH antagonist (22); addition of recombinant LH (31); growth hormone and androgens (29, 32, 33); mild stimulation (3, 18, 29, 22) and double stimulation (34). Madani et al. have suggested that the pregnancy outcomes with the long, short, miniflare agonist and antagonist protocols were similar in PORs (30). However, Filippo et al. have indicated that the antagonist protocol in PORs have advantages over agonist protocols (9). Furthermore a retrospective study (2014) compared minimal stimulation protocol with high-dose stimulation protocol, and their findings showed that clinical pregnancy rate was higher in minimal stimulation group (p < 0.05) (3), but our study showed the similar results for the both protocols. In a retrospective study byYoo et al. (2011), they compared the mild protocol with the conventional stimulation protocol, while their results showed that number of retrieved oocytes, duration of stimulation and gonadotropin dose in mild protocol were lower as compared to those of conventional protocol.Clinical pregnancy rate of the mild stimulation group who were aged over 37 years old was higher but not significant (18). In the first prospective study by Kim et al., they compared the minimal stimulation protocol with the multiple–dose protocol. The results of this study revealed that pregnancy rate and gonadotropin dose applied were the same between both protocols, whereas duration of stimulation and number of oocytes retrieved were lower in the minimal stimulation group (p < 0.001) (35). However, in our study, there were no significant differences regarding duration stimulation and number of oocytes retrievedbetween two groups. A systematic review and meta-analysis by Song Y et al. (2014) reported a significantly lower clinical pregnancy rate in antagonist/letrozole protocol as compared with that of agonist flare-up protocol in PORs (p = 0.001), whereas oocyte retrieved and gonadotropin dose showedno significant differences between two protocols (36). Ravelli et al. (2003) compared the mild stimulation protocol with high-dose gonadotropin in long protocol. Their results showed that the pregnancy rate was similar, but number of oocyte retrieved and gonadotropin dose used were lower in mild stimulation protocol as compared with the related values of high-dose gonadotropin in long protocol (27). Another study (2011) showed that in mild stimulation protocol, thenumber of retrieved oocytes and implantation rate werehigher, whereas clinical pregnancy rate was similar as compared to those of micro-dose agonist protocol (22). Yasa et al. (2013) showed number of oocytes retrieved and gonadotropin dose used were lower in minimal stimulation protocol(p > 0.05).They also showed that pregnancy rate was similar between minimal stimulation and conventional antagonist protocols (39). However, in our study, the number of oocytes retrieved was similar in both groups. Furthermore some studies reported negative effect of clomiphene on the endometrial thickness (37, 38), whereas in our study, there was no significantly difference regarding endometrial thickness between two protocols, suggesting that it was due to early starting clomiphene in cycle.

In present study was shown that IVF outcomes of minimal stimulation protocol were comparable with those of conventional antagonist, except that gonadotropin dose used was significantly lower in minimal stimulation protocol.

The incompatibility between results of previous studies probably originates from differences in design, method sand sample size.

Therefore, minimal stimulation protocol is recommended as an alternative option for conventional high–dose protocol since it is a cost-effective and patient friendly method for PORs.

## Conclusion

This study expressed that minimal stimulation protocol with lower doses gonadotropin and low risk factors provides the similar number of oocytes retrieved and pregnancy rate as compared to the conventional antagonist protocol; therefore, minimal stimulation protocol is likely to be considered as a patient- friendly and cost-effective substitute for PORs.
